# Noncontact Intraocular Pressure Measurement over Bandage Contact Lens and the Effect of Pentacam and Corvis ST's IOP Correction System

**DOI:** 10.1155/2022/4933555

**Published:** 2022-05-09

**Authors:** Xueting Cai, Yi Qin, Sixiu Liu, Zhewei Cheng, Fan Lu, Jia Qu, Ding Chen

**Affiliations:** ^1^Eye Hospital, Wenzhou Medical University, Wenzhou, Zhejiang, China; ^2^Eye Center of the Second Affiliated Hospital, Zhejiang University School of Medicine, Hangzhou, Zhejiang, China

## Abstract

**Purpose:**

To evaluate the influence of intraocular pressure (IOP) measurement while wearing bandage contact lens (BCL) and the effect of Pentacam and Corvis ST's correction systems.

**Methods:**

It was a prospective comparative study. Forty healthy subjects (40 eyes) were included in this study. Eyes were measured using noncontact tonometer (NCT), Corvis ST, and Pentacam before and after wearing BCL. Pentacam's five correction formulas (Ehlers formula, Shah formula, Dresden formula, Kohlhaas formula, Orssengo/Pye formula) and Corvis ST's correction formulas (Ehlers formula and biomechanical corrected formula) were used to correct the IOP values before and after BCL wearing. The IOP values were compared, and the correction effect of different systems were evaluated.

**Results:**

The mean age of the subjects was 24.4 ± 0.60 years. The mean IOP obtained by NCT was 14.8 ± 3.2 mmHg before BCL wearing and was 15.7 ± 3.4 mmHg after BCL wearing. The mean IOP was significantly increased after BCL wearing (0.9 ± 2.9 mmHg, *P*=0.05). Four of the five Pentacam's correction formulas (except Kohlhaas formula) showed no significant difference in the mean corrected IOP values before and after BCL wearing (all *P* > 0.05). The mean IOP obtained by Corvis ST was 13.7 ± 2.8 mmHg before BCL wearing and was 15.0 ± 4.0 mmHg after BCL wearing. The mean IOP was significantly increased after BCL wearing (1.3 ± 2.4 mmHg, *P* < 0.05). Corvis ST's correction formula (Ehlers formula other than biomechanical corrected formula) showed no significant difference in the mean corrected IOP values before and after BCL wearing (*P* > 0.05).

**Conclusion:**

The IOP measurements over BCL by NCT and Corvis ST was found to be increased. The correction systems of Pentacam (Ehlers formula, Shah formula, Dresden formula, and Orssengo/Pye formula) and Corvis ST (Ehlers formula) are useful in correcting the IOP measuring deviation induced by BCL wearing.

## 1. Introduction

Bandage contact lens (BCL) is an effective treatment method for persistent epithelial defects, recurrent corneal erosions, filamentous keratitis, corneal surface irregularities, corneal abrasions, corneal thinning, bullous keratopathy, thermal and chemical burns, and ocular surface reconstruction [1–4]. Intraocular pressure (IOP) is a fundamental and essential ocular parameter in ophthalmological clinics. Frequent measurement of IOP may be needed in some patients during the follow-up. Measuring IOP over BCL is convenient; however, BCL wearing may affect the results of IOP measurement. The frequent removal of BCL for the purpose of accurate IOP measurement may negatively affect corneal epithelization and delay the recovery process. Therefore, correction of IOP to obtain the accurate values is important for BCL wearers in cases where contact lens removal is not desired.

Pentacam is an anterior segment imaging and analyzing system. It has five built-in IOP correction formulas (Ehlers, Shah, Dresden, Kohlhaas, and Orssengo/Pye), which can correct the effects of corneal thickness and corneal curvature of the input IOP values from other tonometers [5]. BCL wearing will increase the apparent corneal thickness measured by Pentacam; however, using Pentacam's built-in IOP correction system to calibrate the IOP values with BCL has not been previously reported. Corvis ST is a new type of noncontact tonometer. It can provide IOP values, corneal thickness, and biomechanical parameters [6]. It can also provide corrected IOP values which is claimed to be less affected by cornea's stiffness [7, 8]. BCL wearing will change the biomechanics of ocular surface, and using Corvis ST to correct the IOP values with BCL has not been previously reported.

In this study, we investigated the influence of silicone hydrogel BCL wearing on the IOP measurement using different tonometries (NCT and Corvis ST) and evaluate the effect of calibration by Pentacam and Corvis ST's IOP correction systems, respectively.

## 2. Methods

### 2.1. Subjects and Materials

This was a prospective comparative study. A total 40 eyes of 40 volunteers (22 males and 18 females) who did not have any ocular or systemic disease were included in the study from January 2019 to December 2019 in the Eye Hospital of Wenzhou Medical University. Institutional Review Board approval was obtained from the Human Research Ethics Committee at Wenzhou Medical University. Informed consent was obtained from all subjects. The study was conducted under the ethical standards outlined in the Declaration of Helsinki.

The inclusion criterion was a normal cornea confirmed with no degeneration or dystrophy after a full ophthalmologic examination. The exclusion criteria were any systemic or ocular disease or any history of ocular surgery. Subjects with corneal astigmatism higher than 2.00 D, and those using soft contact lens in recent 2 weeks or using rigid contact lens in recent 1 month, and those having a history of contact lens intolerance were also excluded. The sample size was decided as follows. We decided that the statistical power was to be 0.80. Thus, to calculate the minimal necessary sample size, we presumed that, between the tonometers, there was a standard deviation (SD) of 2 mmHg, a difference (Δ) of 2 mmHg, a type 1 error (*α*) of 0.01, and type 2 error (*β*) of 0.20. Therefore, the necessary sample size (*N*) = 2(Z*α*/2 + Z*β*)^2^SD^2^/Δ^2^ = 23.4.

The contact lenses used in this study were silicone hydrogel lenses (Sure sight, Alcon, Fort Worth, TX, USA) with 24% water content and 0 D power. The base curvature and the diameter of the lenses were 8.60 mm and 13.80 mm, respectively.

### 2.2. Measurements

A full ophthalmic examination was performed on each subject, including visual acuity measurement and slit lamp biomicroscopy, for anterior and posterior segment evaluation with a 90 D lens. The central corneal thickness (CCT) was measured with Pentacam Scheimpflug imaging system (Oculus Optikgeräte GmbH, Wetzlar, Germany). The instruments used for IOP measurements were a noncontact tonometer (NCT, Topcon CT-80A Computerized Tonometer; Topcon, Tokyo, Japan) and Corvis ST (Oculus Optikgerate GmbH, Wetzlar, Germany). Pentacam's IOP correction system and Corvis ST's IOP correction system were applied to correct IOP values obtained. The IOP correction formulas in Pentacam's and Corvis ST's IOP correction systems are listed as follows:

1. Ehlers formula [9]: ΔIOP = 0.071 × (545* μ*m − CCT)
2. Shah formula [10]: ΔIOP = 0.050 × (550* μ*m − CCT)
3. Resden formula [11]: ΔIOP = 0.040 × (550* μ*m − CCT)
4. Kohlhaas formula [12]: IOP = IOP(measured) + (540 − CCT)/71 + (43 − *K*)/2.7 + 0.75 mmHg; (*K*: corneal curvature)
5. Orssengo/Pye formula [13]: IOP = IOP(measured)/*K*: *K* = (Bc − Cc + C)/B
6. BIOP [7, 14]=(C_CCT1_ × C_CVSIOP_ + C_CCT2_) × C_age_
7. C_CCT1_ = 4.67 × 10^−7^ × CCT^2^ − 7.8 × 10^−4^ × CCT + 0.63
8. C_CCT2_ = −1.73 × 10^−5^ × CCT^2^ + 2.02 × 10^−3^ × CCT − 0.97
9. C_CVSIOP_ = 10 + (CVS − IOP + 1.16)/0.389
10. C_age_ = −2.01 × 10^−5^ × age^2^ + 1.3 × 10^−3^ × age + 1
11. BIOP: biomechanical corrected IOP values by Corvis ST

### 2.3. Procedures

Three different measurements were taken on the right eye of each subject. The first IOP measurement of the naked eyes was implemented by NCT, three consecutive measurement values were made, and the average IOP values were recorded. Then, all of the subjects were examined with Pentacam. Five IOP correction formulas of Pentacam would provide corresponding corrected IOP values. Also, the naked eyes were implemented by Corvis ST, and the IOP and corrected IOP were recorded. After those procedures finished, subjects wore BCLs for 10 minutes and were then measured with these three instruments again. All data were recorded. All of the examinations were carried out between 8:00 am and 10:00 am.

### 2.4. Statistical Analysis

Statistical analysis was performed by Statistical Package for the Social Sciences (SPSS 22.0 for Windows). Paired *t*-test was used to analyze the difference between the IOP values with and without BCLs. The difference between the IOP values of naked eyes' and the corrected IOP values of eye with BCLs was also analyzed. Bland–Altman plots was used to assess the agreement between the IOP values from NCT and Corvis ST. For the Bland–Altman plots, 95% limits of agreement were set as acceptable values. *P* < 0.05 value was accepted as statistically significant.

## 3. Results

**Figure 1:**
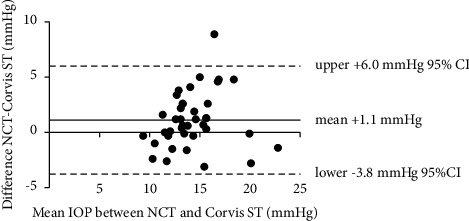
The Bland–Altman plots for the comparison between the IOP values obtained by noncontact tonometer and Corvis ST before BCL wearing. IOP values from noncontact tonometer was higher than corvis ST before BCL wearing (+1.1 mmHg).

**Figure 2:**
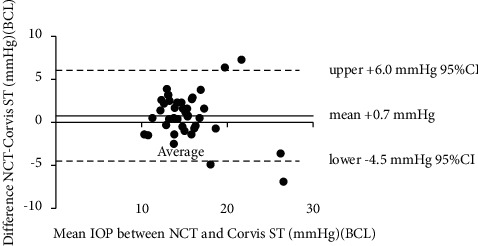
The Bland–Altman plots for the comparison between the IOP values obtained by noncontact tonometer and Corvis ST after BCL wearing. IOP values from noncontact tonometer was higher than Corvis ST after BCL wearing (+0.7 mmHg).

The mean IOP obtained by NCT was 14.8 ± 3.2 mmHg before BCL wearing and was 15.7 ± 3.4 mmHg after BCL wearing. The mean IOP value was significantly higher after BCL wearing (0.9 ± 2.9 mmHg, *P*=0.04). The mean IOP obtained by Corvis ST was 13.7 ± 2.8 mmHg before BCL wearing and was 15.0 ± 4.0 mmHg after BCL wearing. The mean IOP value was significantly higher after BCL wearing (1.3 ± 2.4 mmHg, *P*=0.002) (Table 1). The Bland–Altman plots for the comparison between the IOP values obtained by NCT and Corvis ST before and after BCL wearing are shown in Figures 1 and 2, respectively. Both figures showed the IOP values by NCT was higher than Corvis ST before and after BCL wearing (+1.1 mmHg and +0.7 mmHg, respectively, both *P* < 0.05).

The mean native CCT before BCL wearing was 543 ± 43 *μ*m measured by Pentacam. After BCL wearing, the apparent CCT including the thickness of cornea, BCL and tear film in-between was 555 ± 47 *μ*m. The difference between the native CCT and the apparent CCT was statistically different (*P*=0.01).

Pentacam's five correction formulas (Ehlers, Shah, Dresden, Kohlhaas, and Orssengo/Pye) were used to correct the IOP values obtained by NCT. There was no significant difference between the corrected values of IOP measured before and after BCL wearing (all *P* > 0.05) (Table 1). We also compared the difference between the IOP values obtained by NCT before BCL wearing and the corrected IOP values after BCL wearing by Pentacam's five correction formulas. Only IOP values corrected by formula Kohlhaas's showed significant difference (1.4 ± 2.9 mmHg, *P*=0.004), while the other four formulas (Ehlers, Shah, Dresden, and Orssengo/Pye) showed no significant difference (all *P* > 0.05) (Table 2).

The Corvis ST IOP values were corrected by Ehlers formula and biomechanical formula. The corrected IOP values showed no significant difference before and after BCL wearing (both *P* > 0.05) (Table 1). The difference between the IOP values obtained by the Corvis ST before BCL wearing and the corrected IOP values after BCL wearing was also compared. The biomechanically corrected IOP (BIOP) values showed significantly difference (+1.7 ± 2.1 mmHg, *P* < 0.001), while Corvis ST IOP values corrected by formula Ehlers showed no significant difference (*P*=0.179) (Table 2).

## 4. Discussion

The IOP measurement was overestimated after bandage contact lens (BCL) wearing, either by NCT (0.9 ± 2.9 mmHg) or by Corvis ST (1.3 ± 2.4 mmHg) in this study. This is consistent with previous studies [15–18], while the average difference of IOP before and after contact lens wearing varies among these studies. Previous studies suggested that IOP measurement overestimation with soft contact lenses may be associated with lens power, central thickness, and the rigidity of the lens material [16, 19, 20]. BCL as a special type of soft contact lens may affect the IOP measurement by those factors mentioned above. BCL wearing not only increases of the thickness of the eye wall that is flattened by the air puff of tonometer but also changes the biomechanics of the cornea-BCL complexus.

Previous studies have confirmed that IOP measurement increases when central corneal thickness (CCT) increases. According to Zhang et al. [21] the IOP measured by Goldmann applanation tonometer and NCT increased 0.39 mmHg and 0.64 mmHg, respectively, for each 10 *μ*m increase of CCT. Doughty and Zaman [22] considered that the IOP difference was 3.4 mmHg for every 10% difference in CCT. However, previous studies had different views on the effect of central thickness of soft contact lens on IOP measurement. Ogbuehi [20] considered that the effect of central thickness of soft contact lens with high water content was similar to the effect of central corneal thickness. They also deemed that the influence of high water content lenses with central thickness less than 0.3 mm could be ignored. Nevertheless, McMonnies [15] believed that the influence was significant and nonnegligible when the central thickness of the contact lens was greater than 0.15 mm.

The wearing of BCL increased the apparent CCT measuring values by Pentacam or Corvis ST, which may partially explain the change of IOP measurement values. After BCL wearing, Pentacam indicated that the apparent CCT increased by 12 ± 13 *μ*m; according to Zhang et al. [21], the corresponding increased IOP was about 0.768 mmHg; according to Doughty and Zaman [22], the corresponding increased IOP was about 0.748 mmHg; in our study, the measured IOP values by NCT increased 0.9 ± 2.9 mmHg. After BCL wearing, Corvis ST indicated that apparent CCT increased by 24 ± 15 *μ*m; according to Zhang et al. [21], the corresponding increased IOP was about 1.536 mmHg; according to Doughty and Zaman [22], the corresponding increased IOP was 1.496 mmHg; in our study, the IOP measured values by Corvis ST increased 1.3 ± 2.4 mmHg.

Since BCL wearing increased the apparent CCT values, we may refer to the CCT correction method of IOP to minimize the influence of BCL wearing on IOP measurement. Pentacam's five IOP correction formulas were tried and evaluated, respectively. We compared the corrected IOP after BCL wearing with the uncorrected IOP before BCL wearing and found that the difference of Kohlhaas formula was the most significant. Kohlhaas formula was initially developed to amend the IOP measurement after laser assisted in-situ keratomileusis (LASIK) in which a corneal flap is created, and a modified constant of 0.75 mmHg was added [12]. So, it might not be suitable to use Kohlhaas formula for the correction of IOP after BCL wearing, which would cause overestimation of IOP measurements. Among the other formulas, the difference of Ehlers formula was the smallest, followed by the Orssengo/Pye formula. Therefore, when using a NCT to measure the IOP over BCL wearing, it is recommended to use the Pentacam IOP correction system with Ehlers formula and Orssengo/Pye formula for IOP correction.

Previous studies had shown that the interaction among central thickness, lens power, and elastic modulus of corneal contact lenses could better explain the effect of corneal contact lenses wearing on IOP measurements [17, 23]. Patel and Stevenson [16] studied siloxane hydrogel contact lenses with low water content (24%) and high modulus of elasticity (1.2 MPa) and hydrogel contact lenses with high water content (69%) and low modulus of elasticity (0.91 MPa). They found out that the increase of IOP of low elastic modulus contact lenses was related to the central thickness of corneal contact lenses, and the increase of IOP of high elastic modulus contact lenses was more related to the material. The BCL used in our study was lotrafilcon A silicon hydrogel lens with 24% water content, which is a high elastic modulus lens. Therefore, in addition to the increase of the thickness of corneal contact lens, the elastic modulus of the lens material may also be a factor affecting the measured value of IOP in our study.

The measuring principle of Corvis ST is similar to the ocular response analyzer (ORA; Reichert, Buffalo, NY, USA) [7, 24]. As an air puff is triggered, the cornea deforms inwards up to a first applanation and then into a concave shape. While the air puff decreases, the cornea recovers in the outward direction and undergoes a second applanation before returning to its natural position. The difference between these inward and outward motion applanation pressures is the corneal hysteresis (CH). CH has been considered as an important parameter reflexing corneal viscoelastic property, which is of great relevance in patients with corneal pathologies such as corneal ectasia and keratoconus [25]. ORA takes into account CH and provides a corneal-compensated IOP (IOPcc) which is claimed to be less affected by corneal properties than other tonometric measurements [24]. Mendez-Hernandez et al. compared IOP measurements in keratoconic eyes using five tonometers [25]. They found that IOP measurements by ORA was around 2 to 6 mmHg lower than those by applanation tonometry, which was expected as ORA taking into account low CH in patients with keratoconus.

Similarly, Corvis ST also provides a biomechanically corrected IOP (BIOP) based on IOP correction formula developed by Joda et al. through the analysis of clinical data [7]. It can reduce the influence of corneal elastic modulus and age on IOP measurements by Corvis ST. In fact, the study has found out that the biomechanical correction of Corvis ST measurements is closely related to the CCT, and the BIOP can well eliminate the effect of corneal thickness [14]. In our study, the biomechanically corrected IOP (BIOP) after BCL wearing was significantly lower than the uncorrected IOP before BCL wearing. We speculated BIOP may overcorrected the IOP of eye with BCL. The BCL wearing may affect the accurate measurement of CCT by Corvis ST; therefore, an overcorrection of IOP might happen due to the underlying correction logic of BIOP. In addition, this formula was developed to eliminate the effect of age on the elastic modulus of cornea, but the change of elastic modulus caused by BCL is unrelated to age. Therefore, the BIOP offered by Corvis ST may not be suitable for IOP correction in BCL wearers.

In summary, BCL wearing can overmeasure the IOP, either by NCT or Corvis ST. It was related to the increase of the apparent corneal thickness measurement, as well as the change in the elastic property of cornea-BCL complexus. Certain IOP correction systems have been developed to minimize the effect of BCL wearing on IOP measurement. Pentacam IOP correction system with the Ehlers formula and Orssengo/Pye formula are recommended to correct the IOP values measured by NCT over BCL. Ehlers formula is also recommended when Corvis ST is used to measure the IOP over BCL.

## Figures and Tables

**Table 1:** IOP values before and after BCL wearing (*n* = 40).

IOP (mmHg)	Before BCL wearing	After BCL wearing	*D* value	*P*
IOPt	14.8 ± 3.2	15.7 ± 3.4	−0.9 ± 2.9	0.04
IOPc	13.7 ± 2.8	15.0 ± 4.0	−1.3 ± 2.4	0.002
IOPe	15.0 ± 3.1	15.0 ± 3.3	0.1 ± 2.7	0.87
IOPs	15.2 ± 2.8	15.5 ± 2.9	−0.3 ± 2.7	0.464
IOPd	15.1 ± 2.8	15.5 ± 2.9	−0.4 ± 2.7	0.318
IOPk	15.4 ± 3.0	16.2 ± 3.1	−0.8 ± 2.8	0.072
IOPo	15.1 ± 3.0	15.3 ± 2.9	−0.2 ± 3.0	0.624
IOPce	13.5 ± 2.6	13.0 ± 3.1	0.5 ± 2.5	0.25
BIOP	11.6 ± 2.0	12.0 ± 2.7	−0.4 ± 2.1	0.286

BCL: bandage contact lens; NCT: noncontact tonometer; IOPc: Corvis ST IOP values; IOPt: NCT IOP values; IOPe: NCT IOP values corrected by Ehlers formula; IOPs: NCT IOP values corrected by Shah formula; IOPd: NCT IOP values corrected by Dreaden formula; IOPk: NCT IOP values corrected by Kohlhaas formula; IOPo: NCT IOP values corrected by Orssengo/Pye formula; IOPce: Corvis ST IOP values corrected by Ehlers formula; BIOP: biomechanical corrected IOP values by Corvis ST.

**Table 2:** Comparison between IOP values obtained before BCL wearing and corrected IOP values after BCL wearing (*n* = 40).

	*D* value (mmHg)	*P*
IOPt1–IOPe2	−0.2 ± 4.1	0.736
IOPt1–IOPs2	−0.7 ± 3.5	0.212
IOPt1–IOPd2	−0.7 ± 3.2	0.166
IOPt1–IOPk2	−1.4 ± 2.9	0.004
IOPt1–IOPo2	−0.5 ± 3.3	0.342
IOPc1–IOPce2	+0.7 ± 3.2	0.179
IOPc1–BIOP2	+1.7 ± 2.1	≤0.001

BCL: bandage contact lens; NCT: noncontact tonometer; IOPt1: NCT IOP values before BCL wearing; IOPt2: NCT IOP values after BCL wearing; IOPe2: NCT IOP values after BCL wearing corrected by Ehlers formula; IOPs2: NCT IOP values after BCL wearing corrected by Shah formula; IOPd2: NCT IOP values after BCL wearing corrected by Dresden formula; IOPk2: NCT IOP values after BCL wearing corrected by Kohlhaas formula; IOPo2: NCT IOP values after BCL wearing corrected by Orssengo/Pye formula; IOPc1: Corvis ST IOP values before BCL wearing; IOPc2: Corvis ST IOP values after BCL wearing; IOPce2: Corvis ST IOP values after BCL wearing corrected by Ehlers formula; and BIOP2:biomechanical corrected Corvis ST IOP values after BCL wearing.

## Data Availability

Data are available on request to the corresponding author.
